# Cytomegalovirus (CMV) Polyradiculopathy: An Important "AIDS-Defining" Illness Complication

**DOI:** 10.7759/cureus.58230

**Published:** 2024-04-14

**Authors:** Andrea Finessi, Muhammad A Aziz, Guillermo Izquierdo-Pretel, Adriana Bracho

**Affiliations:** 1 Internal Medicine, Florida International University, Herbert Wertheim College of Medicine, Miami, USA

**Keywords:** cytomegalovirus (cmv), hiv/aids, lower extremity radiculopathy, hiv-associated infection, flaccid paraplegia, cmv encephalitis, polyradiculopathy

## Abstract

We present a case of cytomegalovirus (CMV) polyradiculopathy which occurred concomitantly with CMV encephalitis and CMV retinitis in a patient with HIV/AIDS. Our patient is a 43-year-old male who was admitted with progressive changes in mentation. Cerebrospinal fluid (CSF) analysis showed elevated white blood cell (WBC), low glucose, and elevated protein. The polymerase chain reaction (PCR) panel of CSF was positive for CMV, and other microbiology results were negative. Extensive bilateral CMV retinitis was also noted. The patient was started on ganciclovir and foscarnet, and two weeks after, highly active antiretroviral therapy (HAART) was initiated using Truvada and dolutegravir. The hospital course was complicated by urinary retention and bilateral lower extremity weakness with hypotonia, severe hyperalgesia, and allodynia. An electromyography (EMG) study demonstrated bilateral lumbosacral root dysfunction at L2-S1 with active neurologic changes indicating significant axon loss. Neurology was consulted, and the patient was diagnosed with CMV-induced polyradiculopathy. After three months of treatment, no improvement was noted on lower limbs as he continued with intravenous (IV) ganciclovir. The therapeutic response to induction therapy was discordant as improvement of encephalitis was noted, but not on polyradiculopathy after 180 days of treatment. This highlights the lack of data and treatment guidelines for established CMV polyradiculopathy and not only the necessity for prolonged treatment of CMV polyradiculopathy but also the difficulty in recovery of function once it has developed.

## Introduction

Infection with cytomegalovirus (CMV), also called human herpes virus 5, is contracted hematogenously, transplacentally, and perinatally, via body fluids and transplant organs [1]. It may cause mild mononucleosis-like symptoms sometimes, and like any other *Herpesviridae*, CMV infection persists for the entire lifetime of its hosts. The prevalence of CMV infection in the general US population is about 40-100%, and it is generally asymptomatic in immunocompetent people [1]. However, CMV infection is an important cause of morbidity and mortality in immunocompromised patients, especially the ones with HIV/AIDS with a CD4 count of less than 50 cells/cubic mm. The incidence of CMV neurological infection among individuals with HIV is low (<0.2%), and CMV polyradiculopathy is very rare [1,2]. In this population, CMV reactivation's most common manifestations are retinitis, esophagitis, and colitis, but patients can also experience early symptoms such as fatigue, myalgia, lymphadenopathy, abdominal pain and discomfort, as well as diarrhea. Neurologically, CMV can infect the brain, nerve roots, spinal cord, and peripheral nerves, causing axonal destruction, myelin degeneration, and polymorphonuclear neutrophil (PMN) necrotizing vasculitis of epineural arteries [2]. Within the brain, astrocytes, neurons, oligodendrocytes, and capillary endothelium of nervous tissue can have CMV inclusions [2]. CMV polyradiculopathy in an individual with HIV commonly develops over days to weeks and presents with a progressing ascending neuropathy that includes sensory dysesthesia with radiating pain that starts either in the feet or saddle region, numbness, areflexia, and lower extremity weakness that can lead to bilateral flaccid paralysis or paraplegia [3,4]. There are also associated urinary retention and sphincter dysfunction [3,4]. CMV infection may cause nerve root necrosis, at which point paralysis is irreversible [4].

## Case presentation

Patient information

A 43-year-old male presented with two days of altered mental status, acute kidney injury, and rhabdomyolysis. At the time of admission, he was found to have a CD4 of 22 cells/cubic mm. He has a history of cocaine and fentanyl use disorder and HIV/AIDS of more than 13 years. He has been non-compliant with highly active antiretroviral therapy (HAART) for an unknown amount of time. The acute kidney injury was resolved with intravenous (IV) hydration upon admission. He was found to have CMV encephalitis and retinitis, and after a month of hospitalization, he developed CMV-induced polyradiculopathy. During the course of his stay, his altered mental status improved to AAO×2, and he was able to answer some basic questions. The polyradiculopathy did not improve during his hospital stay. The patient was unemployed and uninsured, but had a strong family support system from his mother, brother, and niece, who have become involved in his care.

Clinical findings

On neurologic exam, the patient showed bilateral lower extremity strength of 0/5 and dysesthesia. He also showed absent ankle, patellar, and Babinski sign bilaterally on the lower extremities. Testing of the upper extremities showed 5/5 strength bilaterally and 2+ radial, bicep, and triceps reflexes bilaterally. In addition, he was AAO×2, provided both relevant answers to simple questions and non-coherent responses, and occasionally spoke to figures who were not in the room. His vision was decreased bilaterally, and a fundoscopic examination showed bilateral retinitis.

Diagnostic assessment and therapeutic intervention

**Figure 1:**
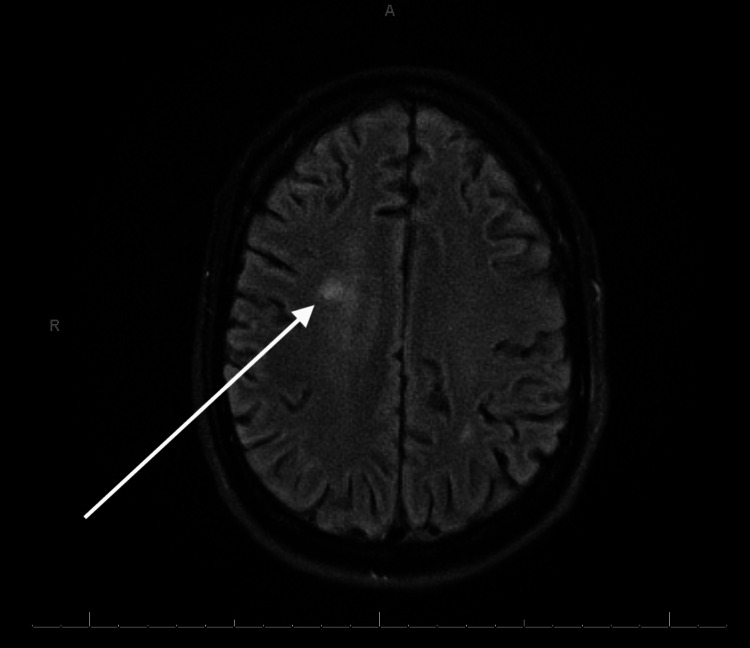
MRI of the brain revealing small acute cortical infarct

**Figure 2:**
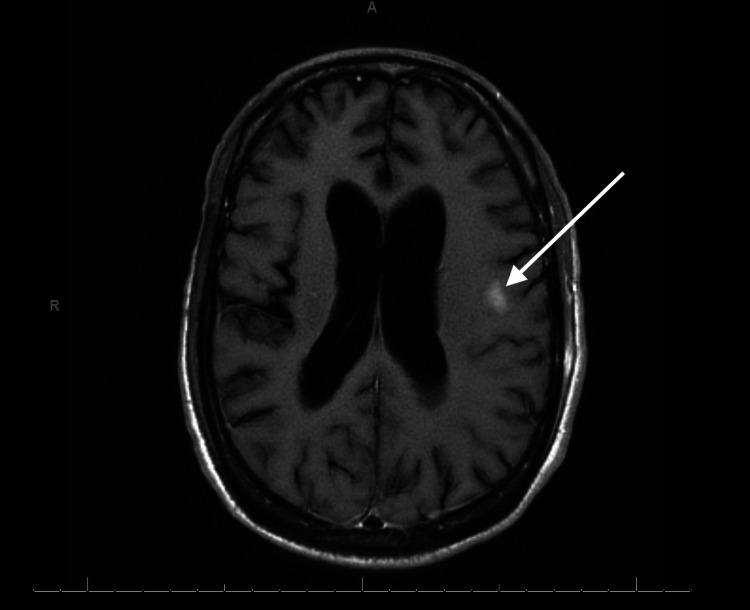
MRI of the brain revealing small acute cortical infarct

**Figure 3:**
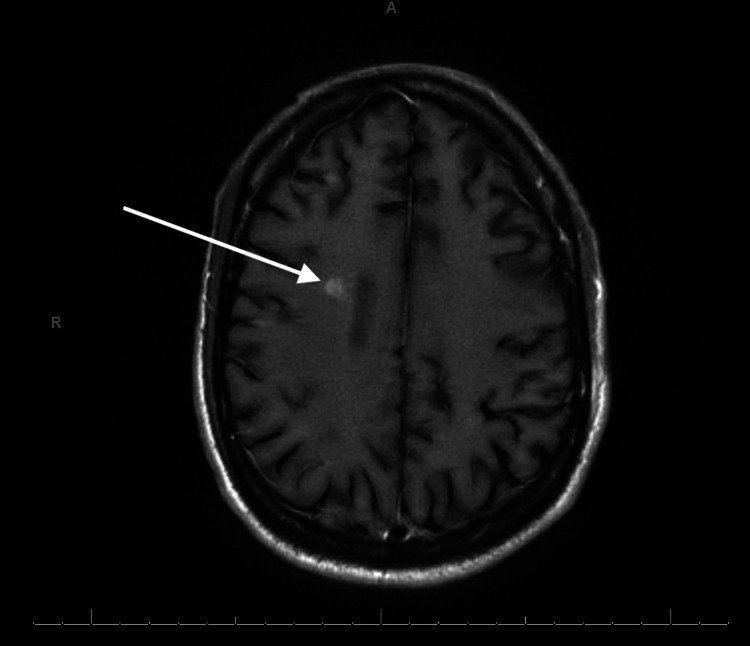
MRI of the brain revealing small acute cortical infarct

The patient presented with an altered mental status that resulted in a workup that included a psychiatric evaluation and a lumbar puncture. Initial tests were inconclusive as to the pathological cause of the encephalitis being from a bacterial or viral origin. The initial cerebrospinal fluid (CSF) fluid analysis showed the following: white blood cell (WBC): 3,280 cells/cubic mm; red blood cell (RBC): 260 cells/cubic mm; glucose: <20 mg/dL; total protein: >600 mg/dL; neutrophils: 88 cells/cubic mm; and lymphocytes: 10 cells/cubic mm, which is more consistent with bacterial infection. Therefore, the patient was originally started on empiric treatment for bacterial meningitis with ceftriaxone, vancomycin, and ampicillin. However, the meningoencephalitis polymerase chain reaction (PCR) panel came back positive only for CMV. Due to the inconclusive results of the CSF fluid analysis and PCR panel, both were repeated. Repeat CSF analysis after 72 hours showed the following: WBC: 1,888 cells/cubic mm; RBC: 862 cells/cubic mm; glucose: <20 mg/dL; total protein: 215 mg/dL; neutrophils: 96 cells/cubic mm; and lymphocytes: 2 cells/cubic mm. Repeat meningoencephalitis PCR panel was again only CMV PCR positive, and other pathogens were negative. Antibiotics were discontinued, and the patient was started on dual IV antiviral treatment with ganciclovir and foscarnet for CMV meningoencephalitis. MRI of the brain revealed multiple small acute cortical infarcts in multiple vascular territories and multiple small acute to subacute lacunar infarcts in the region of the left thalamus. There was also evidence of mild to moderate hydrocephalus with trans-ependymal exudation and mild ependymal enhancement and diffusion, suggesting ventriculitis. In addition, the confluent flair signal intensity in the white matter without signal enhancement may be due to underlying infection (Figures 1-3).

After one month of anti-CMV treatment, the patient's mental status improved to AAO×2, and he was able to follow simple commands and occasionally able to answer simple questions. However, the patient continued to have neurocognitive deficits. Due to the improvement in mental status, he was started on oral valganciclovir, while IV foscarnet and ganciclovir were discontinued. After three weeks on valganciclovir, the patient's mental status had not improved, and he had fluctuating moments of decline. During the initial days of hospitalization, the patient was evaluated by ophthalmology, which discovered extensive bilateral CMV retinitis. He was initially started on atropine 1% eye drops twice a day bilaterally, prednisolone acetate 1% eye drops six times a day on the right eye and four times a day on the left eye, plus weekly ganciclovir/foscarnet intravitreal injections bilaterally. By the eighth week of admission, fundoscopic examination showed stabilization of retinal lesions bilaterally, so weekly ganciclovir/foscarnet intravitreal injections were no longer necessary and were discontinued. Upon presentation to the hospital, the patient's CD4 count was 22 cells/cubic mm, indicating non-compliance with HAART treatment. The patient was started on HAART two weeks after CMV treatment, using Truvada and dolutegravir. Initiation of HAART was delayed by two weeks to decrease the risk of immune reconstitution inflammatory syndrome (IRIS). The patient was also started on atovaquone for PCP prophylaxis.

Follow-up and outcomes

**Figure 4:**
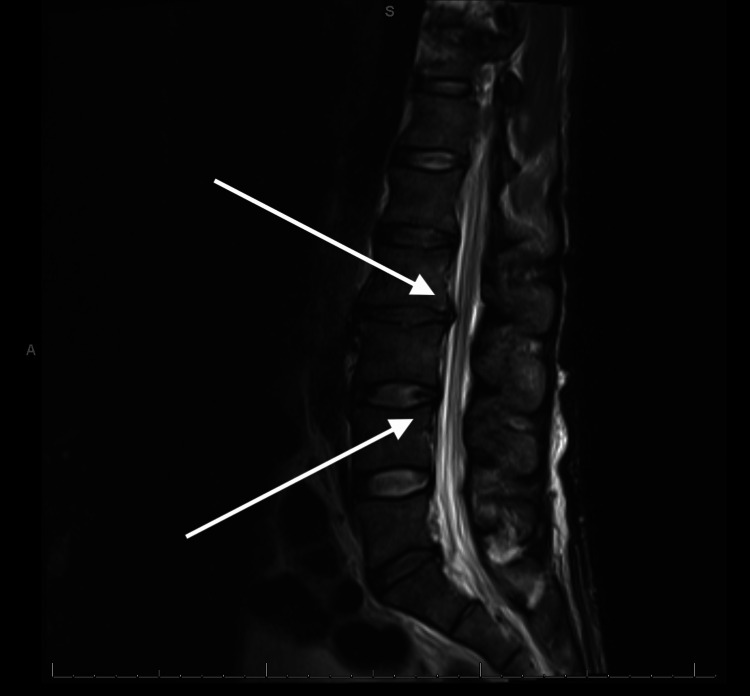
MRI of the lumbar spine showing compression of the spinal cord and spinal canal stenosis

The patient's hospital course was complicated by urinary retention and bilateral lower extremity weakness with hypotonia, severe hyperesthesia, and allodynia. The lower extremities' neurological exam findings were consistent with lower motor neuron injury of the bilateral lower extremities. A lumbar MRI showed moderate to marked spinal canal stenosis at the L2-L3 level, a finding which did not explain symptoms (Figure 4). An electromyography (EMG) study showed bilateral lumbosacral root dysfunction at L2-S1 with active neurologic changes indicating significant axon loss (Tables 1-3). Additionally, underlying peripheral sensory motor axonal neuropathy associated with significant axon loss was observed. Due to the patient's altered mental status and inability to voluntarily move the lower extremities, the possibility of reinnervation could not be evaluated. Neurology was consulted, and on the basis of the EMG and physical findings, the patient was diagnosed with CMV-induced polyradiculopathy. No improvement was seen after 90 days of treatment and after a six-month follow-up visit. At six-month follow-up, the patient was still taking valganciclovir 900 mg/day and antiretroviral therapy with Biktarvy. His CD4 count was 124 cells/cubic mm.

**Table 1:** Sensory NCS Table 1 shows reduced speed and amplitude in the lower extremities during sensory NCS, which demonstrated underlying peripheral sensory axonal neuropathy, resulting in significant axon loss. NCS: nerve conduction study; Lat.: lateral

Nerve/sites	Rec. site	Onset (ms)	NP amp (µV)	Dist (cm)	Vel (m/s)	Temp (°C)
Calf	Lat. malleolus	2.23	1.4	10	44.9	30.7
Lat. leg	Ankle	3.31	4.4	14.8	44.7	30.7

**Table 2:** Motor NCS Table 2 shows the results of the motor NCS, which demonstrated bilateral lumbosacral root dysfunction (L2-S1) with active neurogenic changes that has resulted in significant axon loss. The possibility of reinnervation could not be evaluated as the patient was encephalopathic and did not do any voluntary recruitment. NCS: nerve conduction study; EDB: extensor digitorum brevis; AH: abductor hallucis

Nerve/sites	Rec. site	Lat (ms)	Amp (mV)	Dist (cm)	Vel (m/s)	Temp (°C)	Area (mVms)	Rel area %
Ankle	EDB	4.96	0	-	-	-	0	100
Fibular head	EDB	14.69	0	32.5	33.4	-	0	155
Ankle	AH	5.9	0.4	-	-	-	1	100
Ankle	AH	5.15	0.4	-	-	30.1	1	100
Knee	AH	16.48	0.4	43	37.9	-	0.8	76.7

**Table 3:** EMG summary table Table 3 shows the summary of the EMG study, which demonstrated bilateral lumbosacral root dysfunction (L2-S1) with active neurogenic changes that has resulted in significant axon loss. Additionally, there was underlying peripheral sensory-motor axonal neuropathy which resulted in significant axon loss. EMG: electromyography

EMG summary table			Spontaneous					Recruitment
Muscle	Nerve	Roots	IA	Fib	PSW	Fasc	H.F.	Pattern
R. tibialis anterior	Deep peroneal (fibular)	L4-L5	2+	2+	2+	None	None	No activity
L. iliopsoas	Femoral	L2-L3	2+	2+	2+	None	None	No activity
R. iliopsoas	Femoral	L2-L3	2+	2+	2+	None	None	No activity
L. biceps femoris (long head)	Sciatic (tibial division)	L5-S2	2+	2+	2+	None	None	No activity
R. biceps femoris (long head)	Sciatic (tibial division)	L5-S2	2+	2+	2+	None	None	No activity
L. vastus lateralis	Femoral	L2-L4	2+	2+	2+	None	None	No activity
R. vastus lateralis	Femoral	L2-L4	2+	2+	2+	None	None	No activity
L. tibialis posterior	Tibial	L4-L5	2+	2+	2+	None	None	No activity
R. tibialis posterior	Tibial	L4-L5	2+	2+	2+	None	None	No activity
L. tibialis anterior	Deep peroneal (fibular)	L4-L5	2+	2+	2+	None	None	No activity
L. gastrocnemius (medial head)	Tibial	S1-S2	2+	2+	3+	None	None	No activity
R. gastrocnemius (medial head)	Tibial	S1-S2	2+	2+	2+	None	None	No activity

## Discussion

CMV, also called human herpes virus 5, is a pathogen that is commonly asymptomatic in immunocompetent hosts. CMV infects its hosts for their entire lifetime; therefore, it may reactivate if the host's immune system weakens. For this reason, immunocompromised patients are at higher risk of symptomatic infection, which can manifest as CMV retinitis, pneumonia, and encephalitis. More specifically, up to 40% of AIDS patients with CMV infection will manifest with retinitis, colitis, and encephalitis [5]. Neurological manifestations are less common and include infection to the brain, nerve roots, spinal cord, and peripheral nerves, causing axonal destruction, myelin degeneration, and polymorphonuclear neutrophil (PMN) necrotizing vasculitis of epineural arteries [2]. Within the brain, astrocytes, neurons, oligodendrocytes, and capillary endothelium of nervous tissue can have CMV inclusions [2]. CMV polyradiculopathy in an individual with HIV commonly develops over days to weeks and presents with a progressing ascending neuropathy that includes sensory dysesthesia with radiating pain that starts either in the feet or saddle region with associated numbness, areflexia, and lower extremity weakness that can lead to bilateral flaccid paralysis or paraplegia [3,4]. There may also be associated urinary retention and sphincter dysfunction [3,4].

There are two main theories about CMV neuropathy pathophysiology. The first theory states that CMV affects Schwann cells, macrophages, fibroblasts, endothelium, and neurons by direct membrane fusion, which in turn causes axonal death [6]. At this point, neutrophils get activated and release chemokines and cytokines that amplify the host's response to the virus, causing further inflammation and damage. This may be the reason why on biopsy PMN infiltration and necrosis are noted. The second theory describes that CMV neuropathy is triggered by molecular mimicry. In this process, CMV, during intracellular replication, releases antigens that are expressed on infected cells' surfaces, which are then recognized by antigen-presenting cells, activating lymphocytic response and antibody production. These antibodies cross-react with other cells that express in their surface similar proteins, such as GM2. It is known that fibroblasts infected by CMV produce antigens similar to GM2 and stimulate the production of anti-GM2 antibodies that cause further damage to the neurological system of the infected host [7].

Literature suggests that CMV treatment should consist of antiretroviral therapy such as ganciclovir, foscarnet, and valacyclovir. Treatment should be stopped when repeat CSF PCR is negative for infection. At this point, valacyclovir can be continued as prophylactic therapy [8]. The patient presented with urinary retention and bilateral lower extremity weakness with hypotonia, severe hyperesthesia, and allodynia, consistent with CMV polyradiculopathy. MRI of the brain showed multiple small acute cortical infarcts in multiple vascular territories and a right frontal subacute ischemic process. Confluent fluid-attenuated inversion recovery (FLAIR) signal intensity in the white matter without signal enhancement showed underlying infection, but leptomeningeal disease or infection could not be entirely excluded. CSF studies showed elevated neutrophils, which is more consistent with the polymorphonuclear infiltration theory, as described above.

## Conclusions

This is a patient who developed radiculopathy during his hospital course being treated for CMV encephalitis. Despite adequate treatment of oral valganciclovir and improving mental status, his polyradiculopathy did not improve after six months of treatment. This highlights not only the necessity for prolonged treatment of polyradiculopathy but also the difficulty in recovery of function once CMV polyradiculopathy has developed. Although there is no clear prophylaxis for the development of this complication of CMV infection in AIDS patients, it is imperative that patients who have CMV encephalitis or any other symptom of CMV must be monitored for the subsequent development of polyradiculopathy. In addition, our patient had unexpected CSF findings which led to his delayed diagnosis and treatment of CMV encephalitis. He presented initially with CSF findings suggestive of a bacterial rather than viral infection (increased WBC and neutrophil count with concurrent low glucose); however, these CSF findings are not uncommon for CMV encephalitis. The patient was started on anti-bacterial treatment before the meningoencephalitis PCR panel revealed that he was positive for CMV. Therefore, when an AIDS patient with signs of encephalitis presents with a bacterial infection, a CSF PCR for CMV should be done concurrently as it may present with typical bacterial CSF findings.

## References

[1] Perello R, Vergara A, Monclus E (2019). Cytomegalovirus infection in HIV-infected patients in the era of combination antiretroviral therapy. BMC Infect Dis.

[2] Rutkove SB, Tarulli A, Shefner JM (2022). Polyradiculopathy: spinal stenosis, infectious, carcinomatous, and inflammatory nerve root syndromes. https://medilib.ir/uptodate/show/14149.

[3] Wulff EA, Wang AK, Simpson DM (2000). HIV-associated peripheral neuropathy: epidemiology, pathophysiology and treatment. Drugs.

[4] (2022). AIDS-related cytomegalovirus neurologic disease. https://www.uptodate.com/contents/aids-related-cytomegalovirus-neurologic-disease.

[5] Drew WL (1988). Cytomegalovirus infection in patients with AIDS. J Infect Dis.

[6] Esiri MM, Morris CS, Millard PR (1993). Sensory and sympathetic ganglia in HIV-1 infection: immunocytochemical demonstration of HIV-1 viral antigens, increased MHC class II antigen expression and mild reactive inflammation. J Neurol Sci.

[7] Yuki N (2001). Infectious origins of, and molecular mimicry in, Guillain-Barré and Fisher syndromes. Lancet Infect Dis.

[8] Lowance D, Neumayer HH, Legendre CM (1999). Valacyclovir for the prevention of cytomegalovirus disease after renal transplantation. International Valacyclovir Cytomegalovirus Prophylaxis Transplantation Study Group. N Engl J Med.

